# Gold Nanorods for Light-Based Lung Cancer Theranostics

**DOI:** 10.3390/ijms19113318

**Published:** 2018-10-25

**Authors:** Oscar B. Knights, James R. McLaughlan

**Affiliations:** 1 School of Electronic and Electrical Engineering, University of Leeds, Leeds LS2 9JT, UK; elok@leeds.ac.uk; 2 Leeds Institute of Cancer and Pathology, University of Leeds, Leeds LS9 7TF, UK

**Keywords:** gold nanorod, photoacoustic, photothermal therapy, imaging, theranostics

## Abstract

Gold nanorods (AuNRs) have the potential to be used in photoacoustic (PA) imaging and plasmonic photothermal therapy (PPTT) due to their unique optical properties, biocompatibility, controlled synthesis, and tuneable surface plasmon resonances (SPRs). Conventionally, continuous-wave (CW) lasers are used in PPTT partly due to their small size and low cost. However, if pulsed-wave (PW) lasers could be used to destroy tissue then combined theranostic applications, such as PA-guided PPTT, would be possible using the same laser system and AuNRs. In this study, we present the effects of AuNR size on PA response, PW-PPTT efficacy, and PA imaging in a tissue-mimicking phantom, as a necessary step in the development of AuNRs towards clinical use. At equivalent NP/mL, the PA signal intensity scaled with AuNR size, indicating that overall mass has an effect on PA response, and reinforcing the importance of efficient tumour targeting. Under PW illumination, all AuNRs showed toxicity at a laser fluence below the maximum permissible exposure to skin, with a maximum of 80% cell-death exhibited by the smallest AuNRs, strengthening the feasibility of PW-PPTT. The theranostic potential of PW lasers combined with AuNRs has been demonstrated for application in the lung.

## 1. Introduction

Gold nanoparticles have shown potential for use in biomedical applications such as drug delivery [1,2], biosensing [3,4], molecular imaging [5,6], ultrasound ablation [7] and photothermal therapy [8,9,10]. The combination of unique optical properties [11], relative biocompatibility [12], controlled synthesis [13], and in the case for gold nanorods (AuNRs) a tuneable surface plasmon resonance (SPR) [14], allows them to be effectively translated for many biomedical uses [15].

AuNRs—being rod-shaped—have an advantage over other gold nanoparticles due to the capability of fine control over the peak SPR during synthesis [16]. Their aspect ratio (AR) is a significant factor in determining their SPR, and it enables them to be synthesised with desirable SPRs, particularly in the near-infrared (NIR). As this is a wavelength region that has reduced absorption in biological tissue and enables deeper optical penetration [17,18]. The suitability of AuNRs as contrast agents in optical-based imaging, such as photoacoustic imaging (PAI) [19], and nano-sources of heat in therapeutic modalities, such as plasmonic photothermal therapy (PPTT) [20,21,22], is enhanced with SPRs in the NIR.

Since both PAI and PPTT make use of AuNRs as optical absorbers, it is potentially possible to perform combined diagnostics and therapeutics, commonly referred to as ’theranostics’, with the same AuNR population [23]. A single infusion of AuNRs, that have been functionalised with targeting antibodies, could be administered to a patient and used to identify, then immediately treat, cancerous tissue [24,25]. A current limitation is that these modalities exploit the effects of different laser types [26,27]. PAI is an imaging modality that is dependent on the use of short laser pulses to satisfy the stress confinement conditions and induce a rapid thermal expansion of the AuNRs, resulting in the production of ultrasonic waves [19,28]. Conversely, PPTT commonly utilises continuous-wave (CW) lasers to generate bulk temperature changes in a target region, by continually heating the AuNRs until hyperthermia is achieved [29]. Pulsed-wave (PW) lasers are essential to PAI, and it would be beneficial if PPTT could also be performed using a PW laser since only a single laser system would be needed to achieve the combined theranostic results. The potential for PW lasers to be used for PPTT has been suggested in previous work [30,31,32,33] but analysis of the efficacy of PW lasers under more clinically-relevant parameters is lacking.

Lung cancer is a particularly difficult form of cancer to diagnose and treat, largely due to accessibility issues [34]. An existing technique called ’Endobronchial Ultrasound’ (EBUS) can provide access to a lung tumour from a close proximity to enable the staging of a tumour via needle biopsy [35]. A bronchoscope with a transducer probe located at one end facilitates the imaging of a tumour, and a channel that runs the length of the bronchoscope enables access for various tools, such as a needle [36]. This existing technology could permit a laser fibre to be incorporated into the bronchoscope and allow the laser illumination of a tumour from within the lung. Since space is limited, it would be beneficial if only a single laser fibre was required to achieve both diagnostic and therapeutic effects, indicating the need for PW PPTT.

The successful translation of AuNRs for theranostic applications relies on optimising the AuNRs themselves, as well as the laser source employed. They must exhibit properties that are favourable to both the photoacoustic and photothermal aspects of the technique. Characteristics such as toxicity to healthy tissue [37], PA emission amplitude [38], photothermal conversion efficacy [39,40], cellular targeting and uptake [41,42], and resistance to melting and reshaping [43], all require careful optimisation to achieve an optimal theranostic outcome. One factor that may influence these desired characteristics is the size (or volume) of the AuNRs. It is possible to synthesise many different sized AuNRs that will absorb at a particular wavelength—due to their longitudinal SPR being largely governed by their AR—meaning that a NIR-absorbing AuNR has the potential to be a vast range of sizes [14,44]. Thus, there may exist a particular sized AuNR, with a fixed AR, that exhibits optimal properties for the desired characteristics.

In this work, we consider four different sized AuNRs with similar ARs and SPRs in the NIR (800±26 nm), to determine the optimal AuNR size for theranostic application in the lung. Their size- and concentration-dependent effects on PA response is evaluated, and their capacity to be used as contrast agents for PAI of the lung is examined via the employment of a tissue-mimicking phantom. Finally, the size-dependent PPTT efficacy is determined in a non-small cell lung cancer cell line (A549) using a pulsed laser system as the optical source. The main focus of the presented research is to determine the capability of a single PW laser system, combined with AuNRs of varying sizes but similar SPRs, for the treatment and PAI of lung cancer.

## 2. Results and Discussion

Commercially-bought AuNRs with four different sizes were chosen for this study (see Section 3.1 for details). The motivation behind selecting these AuNRs for investigation was due to their ‘readily-available’ nature (i.e., commercial access), bulk-controllable properties, and scaleable production, which are highly desirable characteristics for clinical translation [45].

**Figure 1:**
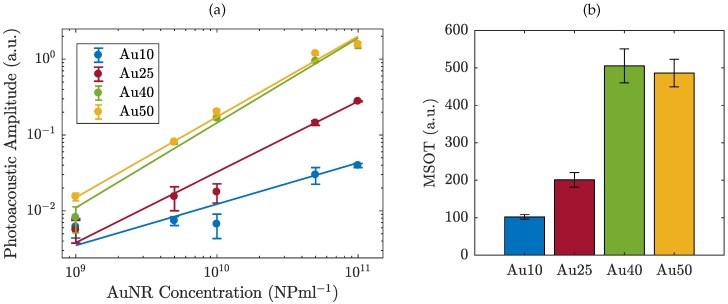
The photoacoustic amplitude of four different sized AuNRs (**a**) as a function of concentration from 1 × 10 ^9^ − 1 × 10^11^ NP/mL as measured with a 1 MHz probe, and (**b**) at a fixed concentration of 1 × 10^11^ NP/mL, measured using a pre-clinical multispectral optoacoustic tomographic (MSOT) system.

The PA response of the four different sized AuNRs was measured across a range of AuNR concentrations from 1 × 10^9^ to 1 × 10^11^ NP/mL and the maximum amplitude calculated (Figure 1a). A logarithmic relationship between AuNR concentration and PA amplitude was observed, indicating the importance of maximising the number of AuNRs delivered to a region in vivo, likely facilitated by the functionalisation and molecular targeting of AuNRs [46]. At equivalent concentrations (NP/mL), the PA signal amplitude scales with AuNRs size, where the larger AuNRs produced the strongest PA signal. Across the entire concentration range, the Au40s and Au50s exhibited similar PA amplitudes. This is likely due to the Au50s only being slightly larger than the Au40s (see Section 3.1) and therefore exhibiting a similar total mass (0.3mg/mL for the Au40s, compared with 0.5mg/mL for the Au50s). At the highest concentration studied (1 × 10^11^ NP/mL) the amplitude of the Au40s and Au50s was almost 40 times larger than the Au10s and 6 times larger than the Au25s, indicating the size-dependent relationship between AuNRs and PA amplitude. As PAI imaging is currently used in the pre-clinical evaluation of light-sensitive nanomedicines [47], a number of commercial systems are available. To give a direct comparison to our laboratory data we tested different sized AuNRs with a pre-clinical multispectral optoacoustic tomography (MSOT) system [48] at a fixed AuNR concentration (1 × 10^11^ NP/mL). Data from both experimental approaches are in agreement (Figure 1b), where the larger AuNRs produced the strongest PA signal at an equivalent number of AuNRs.

**Figure 2:**
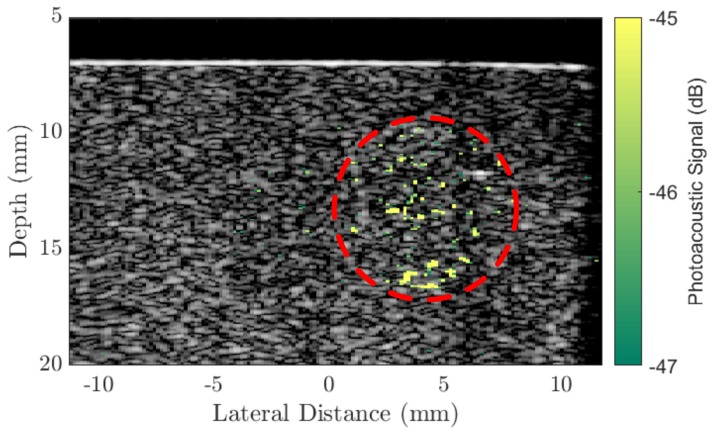
A conventional B-mode plane-wave image (9 compounding angles) of a tissue-mimicking agar phantom with an overlaid photoacoustic image generated by a 8 mm diameter inclusion of Au50 AuNRs (concentration 20 μg/mL) at a depth of 3mm below the surface and a laser fluence =19± 2 mJ/cm^2^ at the surface. The PA signal is referenced to the maximum of the B-mode image (dB). Red circle indicates the region of AuNRs.

Following the study into the PA response of the different sized AuNRs, the largest AuNRs (Au50s) were selected as contrast agents for the PA imaging of a tissue-mimicking phantom (see Section 3.3 for details). The imaging was performed using the Ultrasound Array Research Platform (UARP) II [49]. Figure 2 shows the plane-wave B-mode image of the tissue-mimicking phantom with the PA signal overlaid. Under conventional B-mode imaging, the 9 mm diameter inclusion of AuNRs cannot be seen. However, combined with pulsed laser illumination (laser fluence at the surface =19± 3 mJ/cm^2^, 100 averages) the inclusion is observable. However, the signal within the region of interest is not homogeneous as might be expected. This could be a consequence of the mixing process during the making of the inclusion. The small AuNR inclusions required less than 1 mL of agar and the small quantity of agar mixture meant that it cooled, and set, very rapidly. This did not allow much time to mix the AuNRs into the agar before pouring the mixture into the mould, and while the colour appeared to be uniform, the distribution of AuNRs may not be. Another explanation could be a result of the diminishing photoacoustic conversion efficacy of some of the AuNRs as the continuous chain of laser pulses caused some of the AuNRs in the inclusion to melt. Nevertheless, it is unlikely that a tumour containing targeted AuNRs in vivo would contain an even distribution of AuNRs throughout the tumour volume [50,51].

At a depth of 3 mm below the surface of the phantom, the AuNR-inclusion approximately mimics a typical shallow tumour that may be observed in the lung. Endobronchial Ultrasound (EBUS) [52], is an existing ultrasonic image-guided technique that enables access to tumours within the lung. It is currently used for the staging of lung cancers [53], but could be combined with PA imaging and targeted AuNRs for real-time diagnosis, eliminating the need for needle biopsies [54]. Typically, a bronchoscope for EBUS would contain a channel running the length of the endoscope to allow various tools to be passed through and provide access to the tumour [55]. By replacing this with a laser fibre, illumination of a tumour could be achieved within a close proximity to the tumour, inside the lung. A technique such as the one described could further enable therapeutic applications, such as PPTT since the technique would be identical except for the adjustment of a few parameters such as laser intensity.

**Figure 3:**
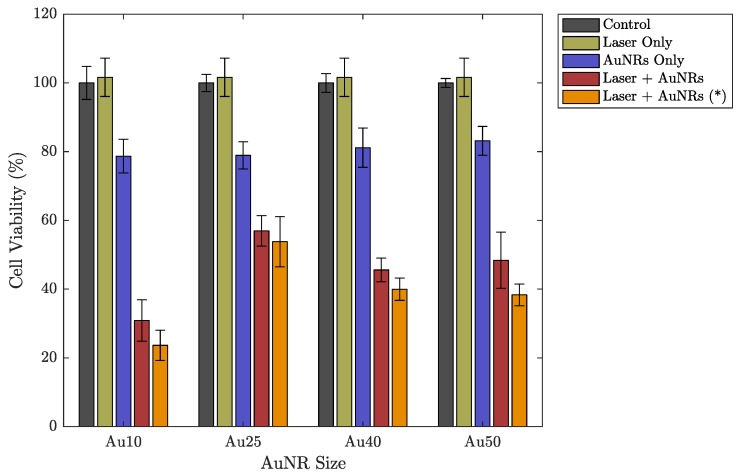
Cell viability of a lung cancer cell line (A549) under different experimental parameters: a control group representing no incubation with AuNRs or laser exposure (grey), after 5 min exposure to laser irradiation without AuNRs (light green), after incubation with different sized AuNRs without laser irradiation (purple), after incubation with different sized AuNRs followed by a 5 min laser exposure (red), after incubation with different sized AuNRs followed by a 5 min laser exposure after replacing the AuNR-media solution with fresh media (orange (*)). Errorbars represent the standard deviation.

**Figure 4:**
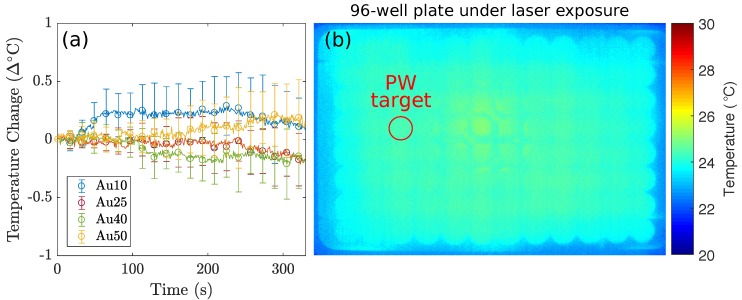
The thermal imaging camera recorded the temperature fluctuations of the 96-well plate containing lung cancer cells incubated with AuNRs. (**a**) The recorded temperature changes of the target well under PW illumination, corresponding to the red circle in (**b**), for 5 min for the Au10s (blue), Au25s (red), Au40s (green), and Au50s (yellow) with the media not replaced, and (**b**) A single frame taken with the thermal camera during the PW-PPTT of lung cancer cells containing AuNRs. The red circle indicates the well under PW laser exposure. The data demonstrates that PW lasers do not induce a bulk temperature change regardless of AuNR size. The observed temperature fluctuations in (**a**) are a result of minute room-temperature changes. Note: Due to the limitations of infrared cameras, the displayed temperature measurements represent the temperature of the plastic bottom of the 96-well plate.

The different sized AuNRs were used as optical-absorbers for the PW-PPTT of a lung cancer cell line (A549) and the PPTT efficacy of the four sizes was considered. The lung cancer cells were exposed to PW laser irradiation after incubation with the different AuNRs, and the laser-induced cell-death was compared depending on if the free-floating AuNRs were removed before laser irradiation or if they were left in the surrounding media (Figure 3). The 5 min laser exposure resulted in a total of 3000 laser pulses (10 HZ PRF), and with a pulse energy of 12±1 mJ (fluence = 19± 2 mJ/cm^2^), the total deposited energy was 36 J. Despite the large deposition of energy, no bulk temperature change was observed for any of the AuNR sizes (see Figure 4). A benefit of no bulk heating in vivo is the reduced damage to healthy tissues from heat diffusion into the surroundings which may be the case if a continuous wave (CW) laser was used [56]. The continuous deposition of energy by a CW laser causes the AuNRs to prolong heating, and the heat dissipates into the surroundings causing a measurable increase in temperature at the target [57]. The laser fluence used in this study was below the guideline maximum permissible exposure (MPE) of skin (31 mJ/cm^2^) [58].

Under PW laser illumination only (i.e., without incubation with AuNRs), it appears that the cells displayed an overall increase in cell viability, compared with the control group (Figure 3). However, this can be explained by the slight variations in the total number of cells seeded in each well at the beginning of the study. The averaged cell viability for each data set is referenced to the control group and therefore if any of the wells contained slightly more cells during the setup process, then it may appear that the laser has promoted growth when in fact it is within the error. Once AuNRs were introduced and combined with PW laser irradiation, significant cell death was observed for all AuNR sizes. Due to the high concentration of AuNRs (17 μg/mL), the AuNRs displayed minimal toxicity on their own (without laser irradiation). Nevertheless, the cells that were exposed to a combination of AuNRs and PW laser irradiation exhibited much greater cell death. The lowest cell viability was produced by the PW laser exposure of AuNRs without replacing the free-floating AuNRs (red bars in Figure 3). While the free-floating AuNRs have not been taken up by the cells, there is an increased number of AuNRs that are situated around the cells and will contribute to the destruction of cell membranes as the laser-light is absorbed. However, the difference compared with the data with the free-floating AuNRs replaced by fresh media was only minimal and within the error. It has been shown that under nanosecond PW irradiation, the location of AuNRs in relation to either the cytoplasm or nucleus of a cell has a minimal effect on PW-PPTT efficacy [31].

**Figure 5:**
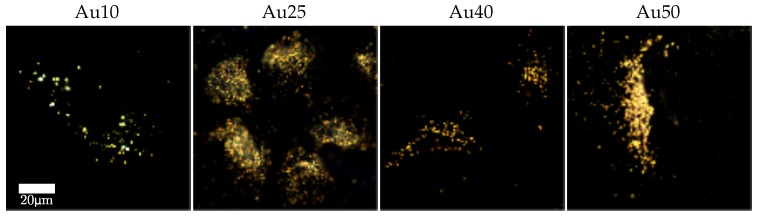
Dark-field images of lung cancer cells (A549) incubated with different sized AuNRs for 24 h at a concentration of 20 μg/mL.

The pathways for cell-death are likely to vary between the two laser types [45]. Figure 5 shows a selection of dark-field images taken of the lung cancer cells (A549) after incubation with the four different sized AuNRs for 24 h. The images show that all four AuNRs have been taken up by the cells and are located predominately within the cytoplasm, as supposed to an even or random distribution as would be the case for non-specific adhesion. The predominant distribution of AuNRs within the cytoplasm for all four AuNR sizes is likely to have resulted in necrosis of the cells by the PW laser, due to the rapid heating of the AuNRs and mechanical stresses induced by the high-intensity laser pulses. Conversely, the slow but continual heating by the CW laser is more likely to have caused the cells to undergo apopotosis, much like traditional hyperthermia [59].

**Figure 6:**
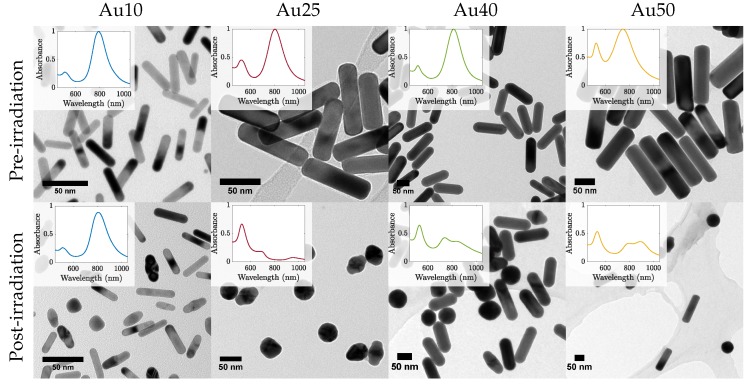
TEM images of all four sizes of AuNRs before (top row) and after (bottom row) pulsed laser exposure with a laser fluence =19± 2 mJ/cm^2^. Inset shows the corresponding measured absorbance spectra.

The most effective PW-PPTT absorbers were the Au10s, where almost 80% of the lung cancer cells were destroyed (Figure 3). The least effective were the Au25s with a maximum reduction of only 54% cell viability, regardless of whether the media was replaced or not. This may be explained by a reduction in optical absorption efficacy as a result of the Au25s melting rapidly under laser illumination (Figure 6) [38]. The Au40s and Au50s also displayed significant melting, resulting in a reduction in PPTT efficacy. However, the Au10s, being the smallest AuNRs considered in the study, displayed the most resistant to melting and could therefore continue to absorb the incident light. It has been shown that smaller AuNRs (<10 nm) are more effective agents for use in PPTT due to a variety of factors including increased cellular uptake, reduced toxicity, and resistance to photothermal degradation [42,60].

The mechanisms that govern photo-degradation of AuNRs has been studied previously [61,62]. It has been shown that AuNR melting occurs at temperatures approximately 40% that of that of bulk gold if maintained at that temperature for extended periods of time. However, when under pulsed-laser illumination, the diffusion of heat to the surroundings is rapid and allows for the AuNR to reach much higher lattice temperatures. The AuNR heating happens within a few picoseconds (i.e., during the nanosecond laser pulse), followed by heat transfer between the absorbing electrons and the lattice (through electron-phonon coupling) [63]. Finally, phonon-phonon coupling results in the cooling of the AuNR as heat is dissipated to the surrounding medium. Under nanosecond-pulsed laser irradiation, the phonon-phonon coupling to the surroundings happens on a comparable time-scale to the laser-induced heating, and therefore the different AuNR sizes will exhibit various heat-dissipation times due to their changing surface area. This may explain why the larger AuNRs (≥25 nm) exhibited a lower thermal stability compared with that of the Au10s since the surface area of the Au10s was large compared with that of the Au40s or Au50s. The larger volume may result in a slower dissipation of heat to the surrounding environment and result in an internal temperature that remains high enough to cause the surface atoms to move [64]. The melting of AuNRs during pulsed-illumination is problematic for prolonged theranostics since their ability to absorb strongly in the NIR is highly dependent on their rod-shape. If melting occurred during imaging or therapy then their capacity to continue absorbing light would be considerably reduced and clinical efficacy would diminish as a result. Their use for theranostic applications would therefore be inadequate.

The size-dependent cellular uptake of the AuNRs may have affected the PW-PPTT efficacy, in addition to the other reasons discussed. However, a large amount of contradicting literature exists surrounding the cellular uptake of AuNRs with regards to aspect ratio, volume, surface chemistry, and cell line [65,66]. Work by Chithrani et al. [41] suggested that as the AR of AuNRs increases, the cellular uptake decreases. However, a more recent study by Kinnear et al. [67] considered the uptake of AuNRs with almost identical properties except AR (AuNR volume, zeta potential, colloidal stability, among others) by an A549 lung cancer cell line. They observed no correlation between AuNR aspect ratio and cellular uptake, below an AR of 7.2 (113×16 nm, SPR ≈1100 nm), suggesting that the AuNRs used in this study may show no size dependence on cellular uptake. However, other parameters such as colloidal stability, particokinetics, monodispersity, etc. will also have an influence on uptake across samples. It is also worth noting that the majority of cellular-uptake studies consider AuNRs with dimensions smaller than the Au40s and Au50s used in this study (typically the longest dimension <100 nm), and therefore their increased size may affect the uptake of these larger particles. In addition to this, each study has other unique factors such as different cell lines, AuNR morphologies, and surface chemistries (such as targeting ligands), making direct comparison difficult. Studies that are able to vary the size of AuNRs while minimising variation in the other properties influencing cellular uptake will be greatly beneficial to current literature.

## 3. Materials and Methods

### 3.1. Selection of Gold Nanorods

AuNRs with four different certified lateral widths (10 nm (Au10s), 25 nm (Au25s), 40 nm (Au40s) and 50 nm (Au50s)) capped with citrate were chosen and bought (Nanopartz, Loveland, CO, USA) for this study. These particular AuNRs were chosen due to their similar ARs and therefore similar SPRs (Au10s: AR = 4.2, SPR = 811 nm; Au25s: AR = 3.7, SPR = 803 nm; Au40s: AR = 3.4, SPR = 813 nm; Au50s: AR = 2.9, SPR = 770 nm). Their size distributions were confirmed via analysis of transmission electron microscope (FEI Technai™F20, Thermo Scientific, Hillsboro, OR, USA) images (examples in Figure 6), and their absorbance spectras via spectrophotometry in previous work [38]. The manufacturer of the AuNRs state that a minimum of 93% of the population of AuNRs were rod-shaped.

### 3.2. Photoacoustic Response of Gold Nanorods

A tuneable pulsed laser system (Surelite™ OPO Plus, Continuum^®^, San Jose, CA, USA) was used to generate a photoacoustic response from the AuNRs by tuning the laser wavelength to the exact SPR of each AuNR sample. The output energy of the laser (pulse width = 7 ns, pulse repetition frequency = 10 Hz, spot size = 5 mm) was increased across a fluence range of 1–40 mJ/cm^2^. Each sample of AuNRs was diluted from their stock solutions to a desired concentration ranging from 1 × 10^9^ NP/mL to 1 × 10^11^ NP/mL before being placed inside an Eppendorf, agitated in an ultrasound bath for 15 min, and irradiated with 20 laser pulses to account for fluctuations in laser energy. The photoacoustic signals were detected using a 1 MHz single-element focussed transducer (V303, Olympus, Hamburg, Germany) mounted on a micrometre translation stage and aligned to the centre of the AuNR solution. The detected signals were recorded with a data acquisition (DAQ) card (M4i.4420x8, Spectrum, Munich, Germany) after a 40 dB amplification (SPA.1411, Spectrum, Munich, Germany).

The amplitude of the recorded photoacoustic signals were calculated by applying the Hilbert transform to a 7 μs windowed region (relating to the diameter of the absorbing region of AuNRs) and integrating across the waveform. Data were also recorded and processed in the same way for a water sample, and a sample with the laser firing but with no output energy. All recordings were repeated three times for reproducibility.

**Figure 7:**
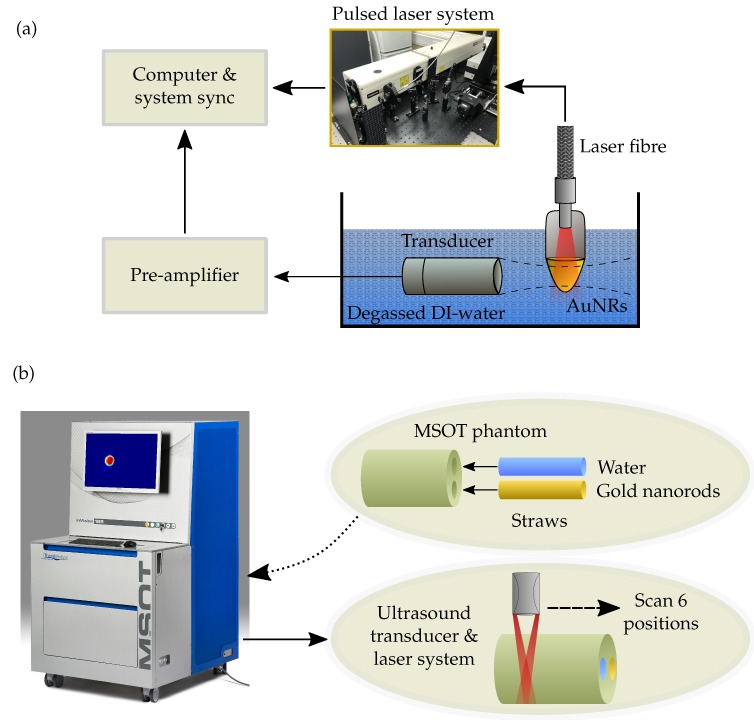
(**a**) Schematic showing the experimental setup for measuring the PA response of the different sized AuNRs at a concentration of 1 × 10^11^ NP/mL. (**b**) Experimental schematic showing a straw containing 1 × 10^11^ NP/mL of AuNRs and another straw containing a water baseline being placed into a typical turbid, agar phantom before a multispectral scan is performed at 6 positions using the MSOT system.

The photoacoustic amplitude of the four AuNRs was also recorded using a preclinical multispectral optoacoustic tomography (MSOT) system (MSOT inVision 128, iThera Medical, Munich, Germany), and Figure 7 shows the experimental schematic. Samples of each AuNR size were made up to a concentration of 1 × 10^11^ NP/mL in a small plastic straw and sealed at each end. The straws containing AuNRs were placed, in turn, inside a typical turbid, agar MSOT phantom, along with a straw containing a water baseline. A multispectral scan (680 nm to 980 nm) was conducted on each sample in steps of 5 nm and the scan was performed at 6 separate locations along the straws for repeatability. The MSOT images were processed and analysed using the viewMSOT software package (viewMSOT 3.8, iThera Medical, Munich, Germany) and the PA amplitude calculated at the peak SPR of each sample.

### 3.3. Photoacoustic Imaging of a Tissue-Mimicking Phantom

An agar-based tissue-mimicking phantom was made based on the work by Rickey et al. [68]. 5.76 g (3% by mass) of agar powder (Acros Organics, Geel, Belgium) was mixed with 132.64 mL filtered, degassed water (82.9% by volume) and 0.8 g of gelatine powder (0.5% by mass). The mixture was heated to 96 °C and maintained at this temperature for approximately 30 min while being continuously degassed. The mixture was left to cool to approximately 70 °C before 1.6 g (1% by mass) of Germall plus (Gracefruit, Stirlingshire, UK) and 16 g Glycerin (10% by mass) was added and mixed. A small amount of the mixture was poured into a separate container where Au50 AuNRs were added to a final concentration of 20 μg/mL and mixed together. This AuNR-agar mixture was poured into a well of a 96-well plate and left to set into a cylindrical shape. Once set, the AuNR-agar cylinder was removed and placed into a larger rectangular plastic container at a distance of 3 mm away from the edge of the container. The rest of the mixture was then carefully poured into the same plastic container and left to set around the AuNR-agar inclusion.

The phantom containing the inclusion of AuNRs was removed once set, and placed inside a tank of degassed, filtered, de-ionised water. A 128-element linear array transducer (L11-4, Verasonics Inc., Kirkland, WA, USA) with a central frequency =7.55 MHZ and -6 dB bandwidth of 90.8% was used in conjunction with the Ultrasound Array Research Platform (UARP) II to image the phantom both actively (plane wave, 9 compounding angles) and passively (photoacoustic signal, 100 averages).

The same PW laser system used in the previous study was also used for the PA imaging of the phantom, with a laser fluence =19± 2 mJ/cm^2^ at the surface of the phantom. The laser Q-switch was synced with the trigger of the UARP II system to enable the passive recording of PA signals generated by the laser. A background signal was also recorded under the same conditions as the photoacoustic signals but with the exception of the laser shutter being closed during recording. This enabled the background noise to be removed from the raw averaged PA data before the images were reconstructed using a delay-and-sum (DAS) beamformer [69]. Finally, the reconstructed PA image was overlaid onto the plane wave B-mode image.

### 3.4. Plasmonic Photothermal Therapy with Gold Nanorods

**Figure 8:**
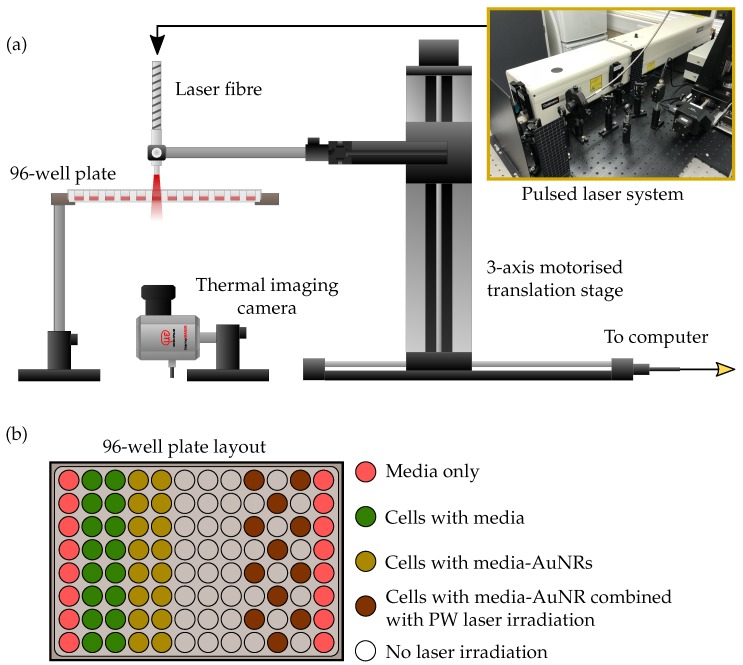
Diagram depicting the PPTT experimental setup. A 96-well plate containing A549 cells with AuNRs is suspended on a platform while a pulsed laser fibre irradiates the wells from above. The fibre is held by a 3-axis motorised translation stage and controlled via a computer interface to scan across the wells. A thermal imaging camera monitors the temperature changes from below. The layout of the 96-well plate is shown above. Wells under laser irradiation were alternated to reduce any potential influences from irradiating adjacent wells.

The same four-sized AuNRs were used for photothermal therapy of a lung cancer cell line (A549, ATCC, Manassas, VA, USA). The non-small cell lung epithelial carcinoma cells were cultured in DMEM (Dulbecco’s Modified Eagle Medium) media supplemented with 10% FBS (Fetal Bovine Serum). Once confluent, a 96-well plate was seeded with 1 × 10^3^ cells per well and incubated for 24 h. The 96-well plate was split into 5 sections (see Figure 8) and the media in 3 of the sections were replaced with a fresh media-AuNR solution at an AuNR concentration of 20 μg/mL while the rest of the columns were replaced with fresh media only. The cells were then incubated for a further 24 h to facilitate the uptake of AuNRs by the lung cancer cells. Immediately before laser irradiation, the media solutions in all of the wells was replaced with fresh media to minimise free-floating AuNRs. In addition, a second 96-well plate was seeded following the same method described above, however the AuNR-media solutions in the wells were not replaced before laser exposure.

The same pulsed laser system used for the photoacoustic study was employed for PPTT of the cells with a laser pulse energy of 12±1 mJ/cm^2^. The laser fibre was mounted onto a 3-axis motorised translation stage to enable the scanning of the fibre tip across the 96-well plate (Figure 8). The cells were irradiated for 5 min from above the plate, while an infrared thermal imaging camera (thermoIMAGER TIM 640, Micro-epsilon-Messtechnik GmbH & Co KG, Ortenburg, Germany) recorded the temperature change of the wells from below, by taking a frame every second for the duration of the exposure. The thermal camera was synchronised with the laser system through the use of an SDK provided by the manufacturer, and this also enabled the direct acquisition of thermal image data into MATLAB for further analysis.

The laser-induced melting of the AuNRs was measured via spectrophotometry and TEM analysis. Samples of AuNRs were exposed to 20 laser pulses with a fluence of =19± 2 mJ/cm^2^ before TEM images were taken and analysed in the open-source software package ImageJ.

Dark-field microscope images were taken of the lung cancer cells (A549) following 24 h incubation with the four different sized AuNRs (concentration = 20 μg/mL) to determine AuNR localisation within the cells.

## 4. Conclusions

We have presented a study comparing the efficacy of four different sized AuNRs for use in PA imaging and PW-PPTT. We observed that generally the larger-sized AuNRs (width <40 nm) produced a stronger PA signal compared with their counterparts (width <25 nm) at equivalent NP/mL. The concentration of AuNRs is a significant factor in PA emissions, as well as AuNR size, indicating the importance of molecular-targeting. The potential for AuNRs to be used as contrast agents in PA imaging of the lung has been demonstrated in a tissue-mimicking phantom. Existing ultrasound-guided imaging techniques such as EBUS can enable access to tumours within the lung and make PA imaging a possibility. For PW-PPTT, the overall efficacy greatly depends on AuNR size in addition to concentration. While the two larger-sized AuNRs (Au40s and Au50s) both induced significant cell death under PW irradiation, we found that the smallest-sized AuNRs studied (Au10s) would be the most suited to inducing cell death with a pulsed laser system at equivalent NP/mL. Since the mass was kept equal across samples, there was a larger number of Au10s incubated with the cells which may have led to an increased number taken up by the cells. This illustrates the importance of mass versus number of particles when considering uptake by cells. If the AuNR samples considered in this study were to be selected for clinical evaluation, we believe the most suitable would be the Au10s, since the highest cell-death using a PW laser was observed, and the PA signal was sufficient to be detectable albeit the total mass of AuNRs was significantly less than the other AuNR samples.

We have shown that it is possible to achieve significant cell-death using a PW laser instead of the conventional approach of using CW lasers, which may facilitate theranostic applications due to the combination of PA imaging and PPTT through the use of a PW laser.
